# 3D in vitro Models of Pathological Skeletal Muscle: Which Cells and Scaffolds to Elect?

**DOI:** 10.3389/fbioe.2022.941623

**Published:** 2022-07-11

**Authors:** Eugenia Carraro, Lucia Rossi, Edoardo Maghin, Marcella Canton, Martina Piccoli

**Affiliations:** ^1^ Istituto di Ricerca Pediatrica Città della Speranza, Padova, Italy; ^2^ Department of Biomedical Sciences, University of Padova, Padova, Italy

**Keywords:** skeletal muscle, skeletal muscle cells, extracellular matrix, myopathies, tissue engineering, three-dimensional pathological muscle models

## Abstract

Skeletal muscle is a fundamental tissue of the human body with great plasticity and adaptation to diseases and injuries. Recreating this tissue *in vitro* helps not only to deepen its functionality, but also to simulate pathophysiological processes. In this review we discuss the generation of human skeletal muscle three-dimensional (3D) models obtained through tissue engineering approaches. First, we present an overview of the most severe myopathies and the two key players involved: the variety of cells composing skeletal muscle tissue and the different components of its extracellular matrix. Then, we discuss the peculiar characteristics among diverse *in vitro* models with a specific focus on cell sources, scaffold composition and formulations, and fabrication techniques. To conclude, we highlight the efficacy of 3D models in mimicking patient-specific myopathies, deepening muscle disease mechanisms or investigating possible therapeutic effects.

## 1 Introduction

Generally, drug discovery, cytotoxicity and pre-clinical treatment are performed and validated in two-dimensional (2D) *in vitro* cell culture systems or *in vivo* in animal models before use in clinical trials. Unfortunately, only a small percentage of drugs or treatments with pre-clinical promising results became approved for clinical practice due to the lack of similarity between these models and the human organism (Hay et al., 2014; Breslin and O'Driscoll, 2013). In the last decades, *in vitro* tissue engineering models have progressed significantly to replicate in the laboratory bench the key aspects of organ or tissue development, regeneration and function (Urbani et al., 2018; Nikolaev et al., 2020). On one hand, tissue engineering is widely employed in regenerating tissues *in vitro* in order to replace *in vivo* those missing or misfunctioning; on the other hand, the same technologies allow to generate three-dimensional (3D) constructs with pathological features, which do not have the purpose of being used as substitute materials, but as models of research and (possibly) diagnostic investigation (Bliley et al., 2021; Nguyen O. T. P. et al., 2021; Borella et al., 2021).

One of the most studied and *in vitro* replicated tissues is the skeletal muscle (SkM), which accounts for approximately 40% of the human body weight. SkM is a tissue largely represented throughout the body, from the facial muscles to the extremities of the limbs. Myopathies, with different genetic origins, can occur in various parts of the body, thus targeting different muscles. Furthermore, since SkM is not only mandatory to perform movements, but it is also involved in metabolic functions, alterations of other organs can determine the malfunctions of the SkM as well. Therefore, due to SkM extension, functions and multiple genetic mutations, it can be affected by various types of damage or defects. The SkM is a complex tissue responsible for voluntary movement, supporting posture and protecting internal organs, but it has also a key role in regulating several metabolic and homoeostatic functions (Noto et al., 2022). Its roles therefore take place: within the tissue itself, preserving the activity of myofibers and their progenitors; restoring the exhausted components due to muscle movements; as well as maintaining specific relationship with other tissues such as the connective, vascular and neural tissues, with which it is intimately connected. For this reason, the study of muscle pathogenesis or the tissue-specific drug screening using simple 2D *in vitro* cultures is highly limited and fails to give a broad overview of all the possible implications external to the muscle tissue itself. Therefore, the need to devise new, more complex and articulated models, which consider not only the use of multiple cell types, but also the impact of the SkM environment and architecture, is becoming increasingly pressing.

In this review, we focus on 3D *in vitro* models of pathological SkM, lingering on different type of cells, scaffolds and fabrication technologies as the up-to-date best options for the generation of reliable research and diagnostic tools.

## 2 Muscle Diseases and Myopathies

Normal SkM function and physiological regeneration depend on several interconnected factors: stem cell activation, myoblast fusion and replacement of old/damaged fibers, myofiber and sarcomere functional integrity, structural and physiological integration within the extracellular matrix (ECM). Each of these components, when impaired or malfunctioning, causes an imbalance of the other parts leading to the manifestation of myopathies that have in common the loss of contractile ability and, therefore, of voluntary movement (Cooper and Head, 2015; Mukund and Subramaniam, 2020).

### 2.1 Inherited and Acquired Muscle Defects

Myopathies are a group of heterogenous pathologies affecting the normal functionality of SkM. According to NIH (NIH, 2022), myopathies are neuromuscular disorders in which muscle fiber dysfunctions lead to muscle weakness. They are generally divided into inherited or acquired diseases, depending on whether they are caused by mutations in genes coding for muscle or neural tissues, or by inflammation, toxic exposition or traumas (Stenzel and Schoser, 2017). Among the inherited myopathies (Supplementary Table S1), Duchenne muscular dystrophy (DMD) is one of the most known and severe disorders, characterized by progressive muscle wasting leading to motor, respiratory and cardiac dysfunctions. DMD is an X-linked disorder affecting 1:5,000 male births and is caused by mutations in the Dystrophin gene that lead to the absence or non-functional production of the protein, essential for SkM contraction and maintenance (Mercuri et al., 2019; Duan et al., 2021). Other mutations in genes that encode specific muscle proteins are responsible for severe inherited muscular dystrophies. Sarcoglycanopathies are a family of rare autosomal recessive muscular diseases which affect striated musculature. They are part of the limb girdle muscle dystrophy (LGMD) family, as they mainly affect proximal muscles of the scapular and pelvic girdles. The pathogenesis of these disorders is very heterogeneous, as is the age of onset, progression and severity (Sandona and Betto, 2009). In general, it is the result of mutations in the Sarcoglycan coding genes, part of the essential Dystrophin-associated protein complex (DAPC). These mutations lead to the weakening of the SkM, which causes difficulty in ambulation and breathing that may evolve into premature death.

Different types of metabolic disorders can have severe repercussions in SkM function, as in the case of Pompe disease, a rare (about 1:40,000 births), inherited and often fatal disorder. It is characterized by a defect in the metabolism of glycogen which accumulates abnormally in the tissues causing a slow and progressive weakening of the muscles (Marion and Paljevic, 2020). This occurs when there is a deficiency in the lysosomal enzyme alpha-acid glucosidase (GAA), which is responsible for the glycogen degradation. The disease affects mainly abdominals (with particular reference to the diaphragm), paraspinals, flexors, adductors and abductors of the hip muscles.

The diaphragmatic muscle is generally one of the most affected muscles in myopathies, and due to its main function in respiration, its involvement frequently leads to dramatic consequences. Congenital diaphragmatic hernia (CDH) is a malformation in which the diaphragm fails to properly form, resulting in the abdominal viscera invasion into the thoracic cavity during embryonic development (Sefton et al., 2018). Despite the moderate incidence (1:3,000 new-born), the etiology of the disease is not completely understood due to the heterogeneous and multifactorial players (Bogenschutz et al., 2020). Indeed, high proportion of CDH cases may be due to new dominant mutations, but epigenetic events could also be a causal factor (Pober et al., 2005). As other abdominal wall defects, CDH is a muscle developmental disease, but diaphragmatic hernia can also appear after traumas in young and adult subjects. In this context, another myopathy known as volumetric muscle loss (VML), is a remarkable disease due to the elevated incidence and the strong social implications, as recently discussed in Testa *et al.* (Testa et al., 2021). VML is an acquired myopathy (Supplementary Table S2) that occurs after trauma or surgery and, as with other muscle defects in which a significant portion of the SkM is missing, often requires regenerative medicine solutions. Recently, other classes of acquired myopathies, for which 3D *in vitro* models can help understanding the development and progression, have been deeply studied and analyzed. Among them, the idiopathic inflammation myopathies, a group of heterogeneous autoimmune disorders, characterized by chronic inflammation of the muscles often accompanied by extra muscular outcomes (Lundberg et al., 2021). Moreover, many drugs and toxic substances such as the use of statins to reduce cholesterol and triglycerides, or the abuse of alcohol, cause myopathy with diverse and sometimes poorly understood pathogenic mechanisms (Pasnoor and Dimachkie, 2019).

In this scenario, considering the actual lack of definitive cures for several types of myopathies, tissue engineering can provide two important solutions: the generation of transplantable muscles, and the production of *in vitro* models to perform tests of therapeutic compounds and better investigate both tissue regeneration and pathology progression.

### 2.2 Affected Cells or Defective Extracellular Matrix

Regardless of the genetic or acquired origin, or whether the disease is a malformation rather than a metabolic dysfunction, the components responsible for the myopathy development and progression are the cells that compose the tissue or the environment in which they live, the ECM.

#### 2.2.1 Skeletal Muscle Cells

*Tissue specific skeletal muscle cells.* The SkM is a finely organized system composed of fused multinucleated myofibers and unique stem cells named satellite cells (SCs) (Mauro, 1961; Relaix et al., 2021). The pool of SCs is localized at the periphery of the myofibers underneath the basal lamina and is responsible for the correct generation, maintenance and repair of SkM. Physiological stresses due to muscle contraction, forced exercise or injuries activate the SCs pool triggering the tissue repair process. SCs reside in a quiescent state; once activated, they enter the cell cycle, providing both self-renew of the SCs pool via symmetric cell division and the restoration of the damaged fibers through the asymmetric cell division mechanism (Evano et al., 2020). Myoblasts, the progeny of SCs, can either fuse with each other forming new myofibers or fuse to existing fibers donating their nucleus to the damaged structures. Some myopathies are extensively correlated to SCs, like progressive congenital myopathy with scoliosis (MYOSCO), occurring upon mutations in the *PAX7* gene, a unique marker of muscle stem cells (Kuang and Rudnicki, 2008). They have been recently discussed in Ganassi *et al.* under the name of ‘primary satellite cell-opathies’, in reference to MYOSCO and ‘secondary satellite cell-opathies’ if the gene mutations affect both SCs and myofibers (Ganassi et al., 2022). According to this definition, muscular dystrophy, congenital, LMNA-related or Emery-Dreifuss dystrophy 2 are secondary satellite cell-opathies, since chronic injury/repair cycles prompt SCs to undergo several cell-cycle divisions affecting the total muscular integrity and functionality.

Despite the central role of SCs in tissue homeostasis, recently, different crucial functions have been attributed to other muscle-resident populations: fibroblasts, pericytes, endothelial cells, neural cells and the cells of the immune system. Together, they are not only responsible for the correct functionality of each muscle, but also for the proper regeneration process that involves the muscles of the body. As for the reparative mechanisms, different cell types can be included in the onset and development of several myopathies.

*Fibroblasts.* Fibroblasts are non-exclusive muscle cells crucial for the SkM maintenance and homeostasis. They are responsible for the structural support, producing the majority of the SkM ECM (Chapman et al., 2016). To date, despite the understanding of the central role of this cell type, great efforts have been made to establish specific parameters for the identification of fibroblast characteristics, therefore different methods, according to cell morphology, localization, ECM deposition and expression of specific markers have been considered. The mostly used markers to identify fibroblast subpopulations are TE7, α-smooth muscle actin (α-SMA), vimentin and transcription factor 4 (TCF4), even though some controversial opinions among scientists dictated from their co-expression with other cell types (Chapman et al., 2016). Fibroblasts are key players in the regeneration process of muscle fibers by entering the cell-cycle soon after tissue damage (Bersini et al., 2018a). Nevertheless, this event needs to be correctly controlled to avoid fibroblast overgrowth during this time. This condition is exacerbated in numerous myopathies and can be the result of acute or chronic injuries, together with a persistent inflammation state, recruiting TGF-β and TNF-α and promoting fibroblastic differentiation into myofibroblasts, thus leading to fibrosis with serious impact on muscle mechanical properties. Indeed, myofibroblasts deposit an uncontrolled and progressive amount of ECM proteins, which replace the normal tissue, ultimately leading to muscle architecture alteration and loss of function (Chellini et al., 2018). Despite fibrosis not being a pathology *per se*, it is a common feature of several myopathies, *in primis* muscular dystrophies.

*Pericytes.* Another cell population, named pericytes, has emerged to contribute to SkM formation, maintenance and regeneration (Dellavalle et al., 2007). Pericytes are mural cells supporting blood vessels. They have been found in different tissues, possessing the ability to differentiate into several cell types, as chondrogenic, adipogenic and muscular cells, contributing specifically in SkM regeneration, fibrosis and fat deposition, as reviewed in detail in Moyle et al. (Moyle et al., 2019). In 2010, Dellavalle and colleagues demonstrated the contribution of pericytes in SkM fiber development and SCs pool maintenance after birth. At the same time, Fuoco et al. observed that pig pericytes, isolated at different ages, possess limited differentiation properties, losing through time the ability to generate muscle fibers and capillaries (Dellavalle et al., 2011; Fuoco et al., 2014). As previously seen for fibroblasts, although DMD and other myopathies make this process complicated, *in vivo* pericytes can be identified by their anatomical localization. Conversely, their identification *in vitro* is more complicated due to the absence of distinctive cell markers, since many of them are shared with SCs or fibroblasts (Uezumi et al., 2010; Sacchetti et al., 2016).

*Telocytes.* Recent and deep analysis of the SkM has uncovered the presence of a new and interesting cell type: telocytes. Telocytes belong to the stromal cell populations and were previously identified in different organs all over the human body before being detected in muscle explants (Popescu and Faussone-Pellegrini, 2010). These peculiar stromal cells are characterized by a small nucleated cell body and a variable number of extremely long processes (named telopodes). Functionally, telocytes are involved in SkM tissue homeostasis, via homo- and heterocellular communications, suggesting a key role in tissue regeneration and repair, especially after traumas. Indeed, they are found mitotically quiescent in healthy SkM (Popescu et al., 2011; Manetti et al., 2019). As seen in previously presented cell populations, the straightforward tagging of telocytes is not yet possible. Nevertheless, the use of a combination of markers such as CD34^+^, CD31^−^, CD117^+^, vimentin, and caveolin-1, or the secretion of VEGF, facilitates the *in vitro* and *in vivo* discrimination from endothelial cells, SCs or fibroblasts. In this context, the potential introduction of telocytes in tissue engineering approaches may help to promote SkM cell proliferation and differentiation, increasing the architectural complexity of *in vitro* muscle models.

*Neural cells.* The nervous tissue is mainly composed of two cell types: neurons and glial cells existing in similar proportions. Neurons are electrically excitable cells, whose main function is to receive, integrate and deliver information to other cells via the axonal connection (Crossman, 2018; Esteves et al., 2020).

Regarding the interconnection with the SkM, each motor neuron (MN) is able to innervate multiple myofibers to form a motor unit, the basic unit of contraction (Buchthal and Schmalbruch, 1980). The signal is transmitted from the axonal terminal branches to specific myofibers by secretion of acetylcholine at the neuromuscular junctions (NMJs). This leads to the depolarization of muscle fibers through Ca^2+^ influx and the generation of a muscle action potential, which results in muscle contraction (Das et al., 2020). Muscle innervation is not only efficient in controlling myofiber contraction, but is fundamental for the communication between muscular and nervous systems contributing to control homeostasis, development, and regeneration of both myofibers and MNs (Raffa et al., 2022). Normal SkM innervation can be altered as a result of surgery, trauma, and neuromuscular diseases. Regardless the cause, the lack of innervation results in loss of muscle functionality, loss of sarcomere organization, atrophy, ultimately leading to the replacement of muscle tissue with fibrous connective tissue (Carlson, 2014).

Neuromuscular diseases are a vast group of pathologies, that can originate either from a disfunction of the neural or the muscular component (Morrison, 2016). An example is spinal muscular atrophy (SMA), an autosomal recessive neuromuscular disease caused by a mutation (usually a deletion) in the *SMN1* gene, which codifies for the Survival Motor Neuron (SMN) protein. This mutation results in a progressive degeneration of the MNs in the anterior horn of the spinal cord, leading to muscle weakness and atrophy with features similar to dystrophic phenotype. The incidence is 1 in 5,000–10,000 live births (Mercuri et al., 2020). The severity of the symptoms is determined by the copy numbers of the centromeric *SMN2* gene, paralogous to *SMN1* and codifying for a shorter (and less functional) version of the SMN protein. Despite SMA involving a defect in a ubiquitously expressed protein known to have a housekeeping role, MNs are selectively vulnerable to SMN deficiency (Park et al., 2010), justifying the muscle weakness as consequence of MN damage. Nevertheless, pre-clinical studies discovered a direct effect of SMN defects on sarcolemma integrity, suggesting the involvement of both MNs and SkM in the pathogenesis of SMA (Cifuentes-Diaz et al., 2001).

*Endothelial cells.* Numerous studies have demonstrated the importance of SkM vascularization to provide oxygen and nutrients, as well as the significance of vascular remodeling to support the developing muscle tissue. The vascular organization is known to be altered in diseased muscle, especially in cases where an extensive regeneration process is involved. Studies have demonstrated that the interaction between muscle and endothelial cells (ECs) goes beyond the simple exchange of oxygen and nutrients. *In vivo*, SCs are usually localized beside vessels, and the number of SCs associated with a myofiber are directly proportional to the abundance of the vascularization in that region (Christov et al., 2007). ECs and SCs have been demonstrated to simultaneously proliferate *in vivo* after damage, and also to cooperate *in vitro*; ECs can stimulate muscle cell growth and migration, while muscle cells exhibit angiogenic-like properties. *In vitro*, myoblasts showed a significant increase in the migration distance when co-cultured with ECs. Furthermore, in this condition, ECs are induced to differentiate and form mature capillaries, indicating a positive effect of myogenic cells on vessel development and maturation (Latroche et al., 2017).

*Macrophages and immune system.* From the immunological perspective, SkM is a unique tissue; its regeneration and damage resolution are highly dependent on the inflammatory response. Generally, inflammatory cells are recruited from the blood stream and specifically targeted to the SkM injured site. Neutrophils and eosinophils arrive first and increase reactive oxygen species (ROS) release to amplify local inflammation. Soon after, monocytes extravasate into the injured area and start the differentiation toward M1-phenotype macrophages, triggering debris removal and SCs activation. Sequentially, the activity of M1 macrophages is replaced by M2-phenotype macrophages, carrying out the final phases of muscle regeneration. Thus, M2 macrophages, together with a series of released cytokines, reduce local inflammation and contribute to the formation of new myofibers (Sciorati et al., 2016; Tidball, 2017). In pathological muscles, as those of patients with neuromuscular dystrophies, metabolic, mitochondrial-related or drug-induced myopathies, there is the coexistence of apoptosis, necrosis and autophagia events often due to the improper M1/M2 dynamics (Yang and Hu, 2018). This chronic and persistent inflammation leads to recurrent macrophage infiltration and heighten proliferation of matrix-producing cells (Duan et al., 2021; Ziemkiewicz et al., 2021), inevitably causing the formation of scars and fibrotic tissues (Greising et al., 2017).

#### 2.2.2 Extracellular Matrix

The ECM plays a fundamental role for cell life in terms of survival, proliferation and differentiation, motility, and communication. Although the ECM is generally composed of water, proteins and polysaccharides, each tissue presents an ECM with a unique composition and topology that is generated during the tissue embryonal development through a dynamic and reciprocal dialogue among the various cellular components (Daley et al., 2008). This unique 3D organization is determinant for each single tissue function. The ECM not only provides a mere structural scaffold for the cells, as it has been considered for a long time, but also contributes to cellular homeostasis and organ functions in an active manner. The ECM bioactive role is carried out by modulating many cellular functions in different ways, likewise, mechanical stimulation, which is achieved through varying the degree of stiffness of the matrix components, directly influences cell differentiation (Mukund and Subramaniam, 2020). This aspect is particularly important in SkM development and regeneration.

The ECM in the SkM plays an integral role in tissue development, structural support, and force transmission (Thomas et al., 2015). The ECM accounts for up to 10% of muscle weight and is organized into three layers: the endomysium that surrounds individual muscle fibers, the perimysium that divides the muscle into fascicles, and the epimysium that provides external support to the entire muscle (Kjaer, 2004). Intramuscular connective tissue is principally composed by type I collagen, a fibrous ECM protein that can vary widely in content and alignment between layers and different muscle types to accommodate function. It is densely layered and highly oriented in the perimysium to optimally transmit force to the tendon.

**FIGURE 1:**
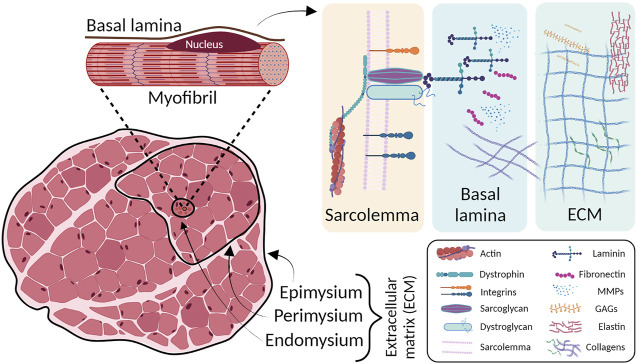
Skeletal muscle ECM layers structure (left) and schematic magnification on ECM principal components (right). The outer layers of connective tissues are divided into epimysium, perimysium and endomysium. Underneath the basal lamina, in close contact with sarcolemma, the ECM major players are located. MMPs: metalloproteinases; GAGs: glycosaminoglycans.

The basement membrane is a specialized layer of ECM that exists between the sarcolemma of each muscle fiber and the surrounding endomysium. The outer basal lamina is mainly composed of type I, III, and VI collagens, whereas the inner basal lamina is composed of nonfibrillar collagen (type IV) and laminin (Kjaer, 2004). Type IV collagen forms a flexible network throughout the basal lamina and connects with laminin near the sarcolemma. Laminin serves as a primary ligand for two sarcolemma receptors: the dystrophin-associated glycoprotein complex and the α7β1 integrin (Grounds et al., 2005). Thus, type IV collagen/laminin/transmembrane receptor complex provides a critical scaffold necessary for lateral transfer of mechanical force from the myofiber to the surrounding connective tissue during contraction. Diminished expression of any of the structures in the basal lamina can severely impair sarcolemma and myofiber integrity (Figure 1).

In this regard, different diseases may affect the SkM ECM. The most common myopathies that affect SkM connective tissue are those involving collagens. Type VI collagen consists of α1, α2, and α3 chains, which are encoded by *COL6A1* and *COL6A2* on chromosome 21q22 and by *COL6A3* on 2q37, respectively (Allamand et al., 2011). Mutations in these genes result in two main muscle disorder types, namely Ullrich congenital muscular dystrophy (UCMD) (severe phenotype) and Bethlem myopathy (minor to moderate phenotype). The UCMD phenotype is caused by loss-of-function mutations, dominant missense mutations or dominant exon-skipping mutations in the *COL6A2* and *COL6A3* genes (Demir et al., 2002; Lampe and Bushby, 2005; Lampe et al., 2008). UCMD is characterized by generalized muscle weakness and striking hypermobility of distal joints. Progressive scoliosis and impairment of respiratory function are typical features (Kirschner, 2013). Bethlem myopathy is a dominantly congenital, comparatively mild disease caused by mutations in the *COL6A1, COL6A2,* or *COL6A3* genes (Lampe and Bushby, 2005; Baker et al., 2007).

Mutations in *LAMA2*, the gene that encodes for the SkM basal lamina isoform, result in a subtype of congenital muscular dystrophy (CMD), LAMA2-MD, characterized by disruption of laminin-211 (Merosin) (Sframeli et al., 2017; Mohassel et al., 2018). LAMA2-MD are classically divided into two main phenotypic categories: a more common, severe, early-onset form, presenting with features of CMD, also known as Merosin Deficient Congenital Muscular Dystrophy type 1A (MDC1A), and a much less common, milder, later-onset form often presenting with a phenotype suggestive of LGMD with prominent joint contractures. Severe LAMA2-MD is one of the most common forms of CMD, accounting for about 1/3 of patients with a diagnosis of CMD (Sarkozy et al., 2020). Muscle biopsies of LAMA2-MD patients show dystrophic changes that can range from mild to severe, and disruption or even absence of laminin-211 can be also observed in the intramuscular motor nerves.

## 3 3D *in vitro* Muscle Models

3D *in vitro* models offer a terrific chance to mimic living tissues and study pathological processes, overstepping the limitations of standard 2D cultures or non-human animal models. In the last decades, many studies have been conducted using different SkM populations and ECM sources and compositions to increase structural complexity and mechanical properties of 3D models. One of the tissue engineering goals in replicating as much as possible the native environment, is to mix different cell populations to recreate specific SkM tissue niches. In this context, the *in vivo* discrimination of different cell types that physiologically compose the tissue plays a key role. The needs to identify new strategies able to distinguish the various cell types is mandatory. Indeed, this aspect, would allow an increased complexity of the *in vitro* models in order to faithfully replicate the *in vivo* environment. In this section we discuss the most recently described 3D models (Supplementary Table S3), considering separately cells and scaffold types.

### 3.1 Cell Sources

Cell source for tissue engineering approaches has always raised ethical and technical concerns due to the collection of human muscle biopsies, especially from severely ill patients. An ideal cell population for SkM tissue engineering should respect defined parameters such as good proliferation rate, massive supply, and ability to terminally differentiate *in vitro*. For the abovementioned reasons, many efforts have been made in the last decades for the maintenance and expansion of human SkM progenitor cell populations (Tamaki et al., 2015; Franzin et al., 2016; Spinazzola and Gussoni, 2017) and for the generation of human induced pluripotent stem (hiPS) cells, the two main categories used in SkM tissue engineering approaches. *In vitro* SC maintenance represents, by logic, one of the biggest pursuits not only because they are the native SkM stem cells, but also because they possess optimal differentiation ability. Specific protocols were set up for the isolation of human SCs. They include mincing steps of the whole muscle, followed by enzymatic digestion and repetitive trituration of the muscle to break both the connective tissue and the myofibers (Romagnoli et al., 2021). Unfortunately, the invasiveness of the collection methods, the variability in the isolation process efficiency (also due to fibroblast contamination), and the reduced myogenic potential after *in vitro* cell expansion have prompted the use of commercially available or biopsy-derived committed myoblasts, impacting on the final generation of *in vitro* muscles depleted in the SCs pool.

In the past, several researchers have focused on the use of adult or fetal stem cells collected from other tissues that possess trans-differentiation properties and assume myogenic capabilities. Through *in vitro* and *in vivo* experiments in pre-clinical models, some works have shown at least a partial ability of these non-resident stem cells to recover muscle damages (Pesce et al., 2003; Negroni et al., 2009; Kawamichi et al., 2010; Huri et al., 2018), but overall none have demonstrated the same myogenic differentiation efficiency that distinguishes SCs. These considerations paved the way for the large use of hiPS-derived cells, with which it is possible to obtain the different cell types composing the SkM, including SCs (Langridge et al., 2021).

iPS cells represent a milestone of bioscience progress (Takahashi and Yamanaka, 2016). These cells are reprogrammed to stay in undifferentiated state by forcing the activation of four mainly transcription factors (*OCT-3/4*, *SOX2*, c*-MYC* and *KLF4*). They can be generated through different strategies following the *in vivo* or *in vitro* necessary requirements. The basic principle is the administration of the Yamanaka factors, allowing the generation of undifferentiated cells ranging from somatic cells obtained by non-invasive procedures such as urine and blood collection to skin biopsies (Takahashi et al., 2007). The differentiation of iPS cells into myogenic cells follows a multistep process recapitulating the embryonic development. Embryologically, SkM tissue originates from the mesoderm together with cardiac muscle, connective tissue, bones, blood cells and kidneys. Therefore, all the myogenic progenitors in the body originate from the somites of the mesoderm which, upon specific signals, start to express muscle-specific myogenic factors known as myogenic regulatory factors (*MYOD*, *MYF5*, *MYOGENIN* and *MYF6*). The obtained myoblasts can fuse together and form muscle fibers (Chal and Pourquie, 2017). This cascade process is recreated *in vitro* to induce SkM cell generation from iPS cells. Usually, this induction is performed by overexpressing the myogenic transcription factors *MYOD* or *PAX7* (Darabi et al., 2012; Goudenege et al., 2012), or alternatively using recombinant proteins or small molecules (Borchin et al., 2013; Chal et al., 2016; Hicks et al., 2018). Importantly, *MYOD* has been reported as the master regulator of myogenic differentiation since its overexpression is sufficient to force non-myogenic cells into myogenic fate. This notion is further supported by evidence in mouse models in which *MYOD* depletion impacts on muscle development and regeneration (Yamamoto et al., 2018). To date, the first evidence of how *MYOD* overexpression leads to SkM cell generation dates back to the 90s (Dekel et al., 1992). The researchers applied this method not only to obtain healthy, but also myopathic iPS cells, such as those derived from DMD and Pompe diseased patients (Abujarour et al., 2014; Shoji et al., 2015; Sato et al., 2017; Yoshida et al., 2017).

The most used technique for *MYOD* overexpression has been published by Tanaka and co-workers, using a *Piggybac* transposon system, allowing cDNA to stably integrate and ensuring efficient gene expression (Tanaka et al., 2013). Notably, other groups used the same strategy, generating cell populations able to recreate specific disease characteristics such as the absence of dystrophin, increased creatine kinase release or altered calcium influx for DMD (Albini et al., 2013; Shoji et al., 2015; Monfoulet et al., 2021).

Another example is the mesangioblast-like stem cells obtained from iPS cells that are capable of differentiating to SkM cells upon forced *MYOD* expression (Maffioletti et al., 2015). Even though *MYOD* overexpression is the most used strategy to drive SkM differentiation, it forces cell-cycle to arrest, not allowing the generation of SCs, thus precluding an accurate recapitulation of SkM niches *in vitro*. In this context, inducing the generation of SkM cells by forcing the expression of *PAX3* or *PAX7* during development prior to *MYOD* activation permits to get closer to the real *in vivo* SkM environment with the *in vitro* generation of both myofibers and stem cells. Moreover, the use of small molecules and other factors might further overcome this issue, paving the way for safer therapeutic applications. A demonstration of this concept was published in 2016 by Chal *et al.*, that set up a protocol for the generation of *PAX7* positive cells from hiPS cells (Chal et al., 2016) due to the dual modulation of Wnt and bone morphogenetic protein (BMP) pathways. These two pathways allow the bypassing of genetic manipulation and cell sorting, two processes that generally characterize other protocols. In this regard, Rao *et al.* have succeeded with the production of 3D bundles via transient expression of *PAX7* in cells differentiated from hiPS cells. Over a month of culture, the 3D muscles increased in structural and molecular maturation. In addition, upon implantation, the 3D constructs displayed progressive vascularization (Rao et al., 2018). Increasing the complexity, Maffioletti and co-workers demonstrated, in an elegant model, the possibility to generate artificial SkM from hiPS cells combining, together with muscle cells, other isogenic cell populations, such as endothelial cells, pericytes and neural progenitors, generating a platform that could bring together regenerative medicine and drug development under the same translational technology (Maffioletti et al., 2018).

### 3.2 Type of Scaffold

The innovative and multidisciplinary strategies adopted in the recent years to rebuild *in vitro* SkM tissue architecture and complexity can be a game changer. Nevertheless, multiple combinations of different scaffolds, techniques and approaches are very broad and still not well established. In this section, we describe the most commonly used scaffolds and the relative fabrication strategies adopted to model *in vitro* SkM tissue.

The scaffold is defined as the accommodating material that enhance and promote mechanical support to enable cell engraftment, spatially guide tissue organization, and sustain cell proliferation, differentiation, and final maturation. Nowadays, the main scaffold types for SkM tissue engineering are based on synthetic-derived, natural-derived materials, and a variety of different combinations of hydrogels and ECM peptides that aim to mimic the environmental cues of the natural ECM, or to act like instructive materials able to induce specific cell responses (i.e. Liquid Crystalline Networks promoting myotubes fusion and differentiation) (Martella et al., 2021). Synthetic-derived scaffolds have the advantage that their mechanical and structural properties can be customized with extreme precision according to the final application. Specifically, synthetic or chemical scaffolding techniques are mainly composed of poly-L-lactic-acid (PLLA) and polylactic-co-glycolic-acid (PLGA), polyurethane (PU), polyethylene glycol (PEG), and polyvinyl alcohol (PVA) (Boso et al., 2020). These materials can be tuned in a precise and defined manner to induce or promote the formation of organized and mature tissues. Moreover, they can be loaded with specific bioactive molecules and growth factors to induce cellular and tissue stimulation (Maleiner et al., 2018). Although these materials present many advantages in terms of manufacturing, synthetic scaffolds lack the biochemical components that are indispensable for cellular behavior guidance which make the scaffold a peculiar environment for the function that the tissue should perform. From this perspective, natural-derived materials have an intrinsic affinity with the physiological environment. In recent years, they have been widely used as both plate-coating for 2D cultures (Serena et al., 2010) and scaffolds for the *in vitro*-grown tissues (Urbani et al., 2018). Organ- and tissue-donor decellularization is one of the most used techniques to obtain tissue-specific and well preserved natural-derived scaffolds for the generation of 3D constructs (Mayorca-Guiliani et al., 2019). Indeed, this approach, allows the preservation of a variety of resident ECM components, maintaining at the same time 3D tissue architecture and ultrastructure (Gerli et al., 2018), features that exert great influence on cellular behavior. All these considerations assume a relevant importance when dealing with muscle tissue, where the myofiber bundle organization and the biomechanical characteristics make this organ a hardly replicable structure with artificial systems. Decellularized scaffolds were employed for the *in vitro* generation of both linear (Urciuolo et al., 2018) and non-linear muscles (Trevisan et al., 2019), demonstrating to sustain and support *in vitro* and *in vivo* cell engraftment, proliferation and maturation of engineered constructs (Jank et al., 2015).

Synthetic, natural derived and combined scaffolds are frequently used as hydrogel formulation. Hydrogels are a family of hydrophilic polymers with high water content and, due to their liquid state, are commonly used to entrap the cells together with biomolecules such as growth factors (Langridge et al., 2021). As described before, the main ECM components are collagens and laminin. Overall, the most common hydrogel-based strategies aim at recapitulating one or more aspects of the SkM ECM such as stiffness, patterning, and biochemical compositions. The combination of collagen-derived hydrogel with Matrigel or fibrin has been demonstrated to produce reliable models that closely recapitulate important aspects of the human SkM physiology and metabolism (Khodabukus and Baar, 2016). For instance, Gholobova and colleagues presented the production of human bio-artificial muscles, named BAM, based on fibrin-derived hydrogel casting procedure. The authors showed a tissue engineered model useful for intramuscular injection studies, for drug testing and screening (Gholobova et al., 2018). More recently, Khodabukus *et al.* described the use of 3D engineered muscle tissues (myobundles) formed by mixing cells, Matrigel and fibrinogen. The casting technique they employed was aimed at forming the supporting hydrogel scaffold to provide an *in vitro* platform to: establish patient-specific drug-induced myotoxicity test; assess novel injury biomarkers; and guide preclinical and clinical studies (Khodabukus et al., 2020). Furrer *et al.* showed how Matrigel scaffolding can be combined with the 3D bioprinting technologies in order to reproduce reliable and standardize protocols to obtain human SkM models, recapitulating exercise and pharmacological responses (Alave Reyes-Furrer et al., 2021). In this research field, the new trend is the use of tissue specific ECM-derived hydrogels based on SkM decellularized ECM as reported by several authors (Choi et al., 2016; Lee et al., 2020). Decellularized ECM has emerged as novel natural-derived hydrogel for muscle tissue engineering because, compared with other hydrogels, even after the decellularization procedures, most of the obtained formulations still contain growth factors, cytokines, proteoglycans, and structural adhesive proteins, which represent tissue-specific biochemical cues. Our group recently described the production of a decellularized ECM-derived hydrogel obtained from porcine diaphragms, representing a useful biological tool for SkM tissue engineering applications, as it is capable of accommodating muscle cells and also of modifying its own biomechanical characteristics following concentration increase or crosslinking.

### 3.3 3D Model Fabrication Technologies

Several approaches have been developed for SkM regeneration according to their application. They can generally be classified in three main categories: *in vitro, in vivo* and *in situ* methods. Each approach has advantages and limitations that determine and circumscribe their use. Importantly, in this context, it is not guarantee that *in vitro* models, characterized by environmental complexity and functional capabilities, can then be easily adapted for *in vivo* application. The translation of 3D models can be affected by scalabilities, especially by the huge cell amount required to obtain bundled myofibers for *in vivo* applications (i.e. for human VML treatment). Moreover, the biomaterials used to mimic ECM and build tissue structure should be bio-compatible with the final recipient (Qazi et al., 2015; Smoak and Mikos, 2020). In the following paragraphs, we focus especially on *in vitro* models, citing only the most prominent examples of *in vivo* or *in situ* application.

For exclusive *in vitro* purposes, in recent years the work belonging to the group of Professor Gilbert has been of great relevance. They reported a system designed to produce 3D SkM microtissues, using immortalized myoblast lines from healthy and DMD donors, able to self-organize following electrical stimulation (Ebrahimi et al., 2021). This work was preceded by a game-changer paper that filled the lack of an easy-to-use platform for the fabrication of micro-SkM models with high reproducible physiological responses and exhibiting macro-tissue characteristics. The 3D models were generated in reusable 96 multi-well plates using primary human myoblasts directly obtained from muscle biopsies (Afshar et al., 2020).

Generally, the strategies to obtain 3D SkM models rely on self-organized 3D constructs or organoid-like cultures (Li et al., 2011; Takahashi et al., 2013; Takahashi and Okano, 2015; Shin et al., 2022), and scaffold-based 3D models (Jalal et al., 2021) even though the majority of protocols require the presence of scaffolds which can be of different composition and formulation. Currently, cells are embedded in Matrigel, collagen or fibrin hydrogels and the 3D constructs take place due to the presence of two attaching points able to provide proper physical cues as the tensile strength (Ostrovidov et al., 2014; Gholobova et al., 2018; Laternser et al., 2018; Maffioletti et al., 2018; Afshar Bakooshli et al., 2019; Capel et al., 2019; Afshar et al., 2020; Fleming et al., 2020; Rajabian et al., 2021). In these cited papers, hydrogels are manually loaded. However, together with the presence of attaching points, other techniques have been used such as scaffold topography, including micropatterned substrates, to better mimic ECM architecture and mechanical properties, and to guide muscle cell differentiation (reviewed by (Boso et al., 2020)).

To overcome the limitations associated with the manual production of the above-mentioned 3D models, 3D bioprinting technology became more relevant due to the feasibility of finely mimic SkM tissue complexity. The layer-by-layer stratification leads to the deposition of cells and ECM components with high precision and accuracy; recreating complex micro-geometries and mechanical cues. The fidelity of the process depends on several parameters such as the biocompatibility, viscosity, and gelation properties of the synthetic and biologic inks which should be modulated to preserve cell viability. Thus, the inks should provide cell homogeneous distribution, avoiding cell clusters and aggregates formation (Ferris et al., 2013). The most used 3D bioprinting approaches are based on extrusion method; this means that sheer forces can act on cells and inks possibly modifying cell viability and ECM component properties (Jessop et al., 2017). In 2018 Kim and colleagues developed a 3D bioprinting strategy for the generation of implantable muscle using human primary muscle progenitor cells. The bioprinted constructs were able to integrate *in vivo* and form networks with host vascular and neural tissues in a rodent model. Technically, the constructs were composed of three layers: a hydrogel bioink with embedded cells; a sacrificial acellular gelatin bioink; and a thermoplastic polymeric (PLC) pillar supporting structure. For their production, multiple strips of the bioinks were deposited in parallel in the PLC structure to control cell viability, differentiation, orientation, and oxygen and nutrient diffusion. The authors compared the results of bioprinted and non-printed constructs containing the same cell density. Interestingly, the data showed significant differences, over time, in cell viability, differentiation and proper alignment in favor of the bioprinting approach (Kim et al., 2018). Recently, an incredibly innovative research performed by Urciuolo and colleagues demonstrated the possibility of intravital 3D bioprinting of muscle-derived stem cells and 7-Hydroxycoumarin-3-carboxylate (HCC)-gelatin hydrogel under the epimysium of hindlimb muscle in mice that leads *de novo* formation of myofibers (Urciuolo et al., 2020). Other groups also confirmed the great potential of 3D bioprinting approach: Choi *et al.* developed a strategy based on the use of a tissue-derived bioink able to maintain a high cell viability, proper oxygen tensions and *de novo* muscle formation inside the printed structures upon *in vivo* implantation (Choi et al., 2019).

The use of cell-embedded hydrogels highlights the self-organization properties of the different cells composing SkM tissue for the generation of *in vitro* differentiated muscles. However, SkM is a highly mechanically active tissue characterized by rigorous physiological stiffnesses, able to vary according to the developmental stage, physiological status, traumas or injury recovery (Gosselin et al., 1998; Wood et al., 2014; Lacraz et al., 2015). Thus, the possibility to modulate these mechanical aspects represents a crucial point. 3D structure biomechanical properties can be modified not only by controlling hydrogel stiffness (i.e. with different kind of crosslinking) but also by substituting the use of hydrogel with decellularized scaffolds which intrinsically recapitulate the anatomy and the biological composition of SkM ECM. The main difference in the use of decellularized ECM instead of hydrogels concerns the recellularization process: cells are no longer embedded in a liquid solution but need to be injected inside the solid scaffold. This can be achieved, for example, through the perfusion method exploiting the native decellularized vascular tree (Jank et al., 2015), or directly inserted in several points through the use of small stings. Our group demonstrated the possibility to obtain mature and metabolically active 3D constructs by injecting human SkM primary cells inside murine decellularized diaphragmatic muscle (Trevisan et al., 2019). These results were recently confirmed and implemented by introducing the use of custom-made bioreactor able to mimic *in vitro* the physiological muscle movements leading to the generation of more mature and physiologically aligned structures (Maghin et al., 2022).

Besides the choice of the proper cells and materials in the generation of 3D SkM models a key role is reserved to the external stimuli. Given the high reactivity of the cells, by modifying the microenvironment and thus acting on the ECM compartment, it is possible to trigger or increase proliferation, maturation, and alignment of SkM cells. In the recent decades, several papers have been published describing the efforts to build valid supports and devices for SkM stimulation varying from deformable membranes to proper bioreactors. Generally, these papers present custom-built systems affecting the comparability of different studies. Nevertheless, the main principle is to recapitulate *in vitro* the passive muscle development and growth (ramp stimulation) and the muscle training and exercise (cyclic stimulation). Using different biomaterials with human SkM cells and applying diverse magnitude strains and frequencies, numerous groups demonstrated the positive effects of mechanical stimulation for the generation of reliable 3D *in vitro* models. This is confirmed by the spectrum of analyzed outcomes as promotion of ECM remodeling (Auluck et al., 2005; Maghin et al., 2022), increased SkM fibers dimension (Powell et al., 2002; Juhas et al., 2016) and alignment (Matsumoto et al., 2007) or enhanced SCs activation (Fleming et al., 2020). Together with mechanical strain, other kinds of exogenous stimuli such as electrical stimulation, can be added to the 3D muscle constructs (Rangarajan et al., 2014; Khodabukus et al., 2019).

## 4 Efficacy of 3D Pathological Models in Deepening Muscle Disease Mechanisms or Investigating Therapeutic Effects

**FIGURE 2:**
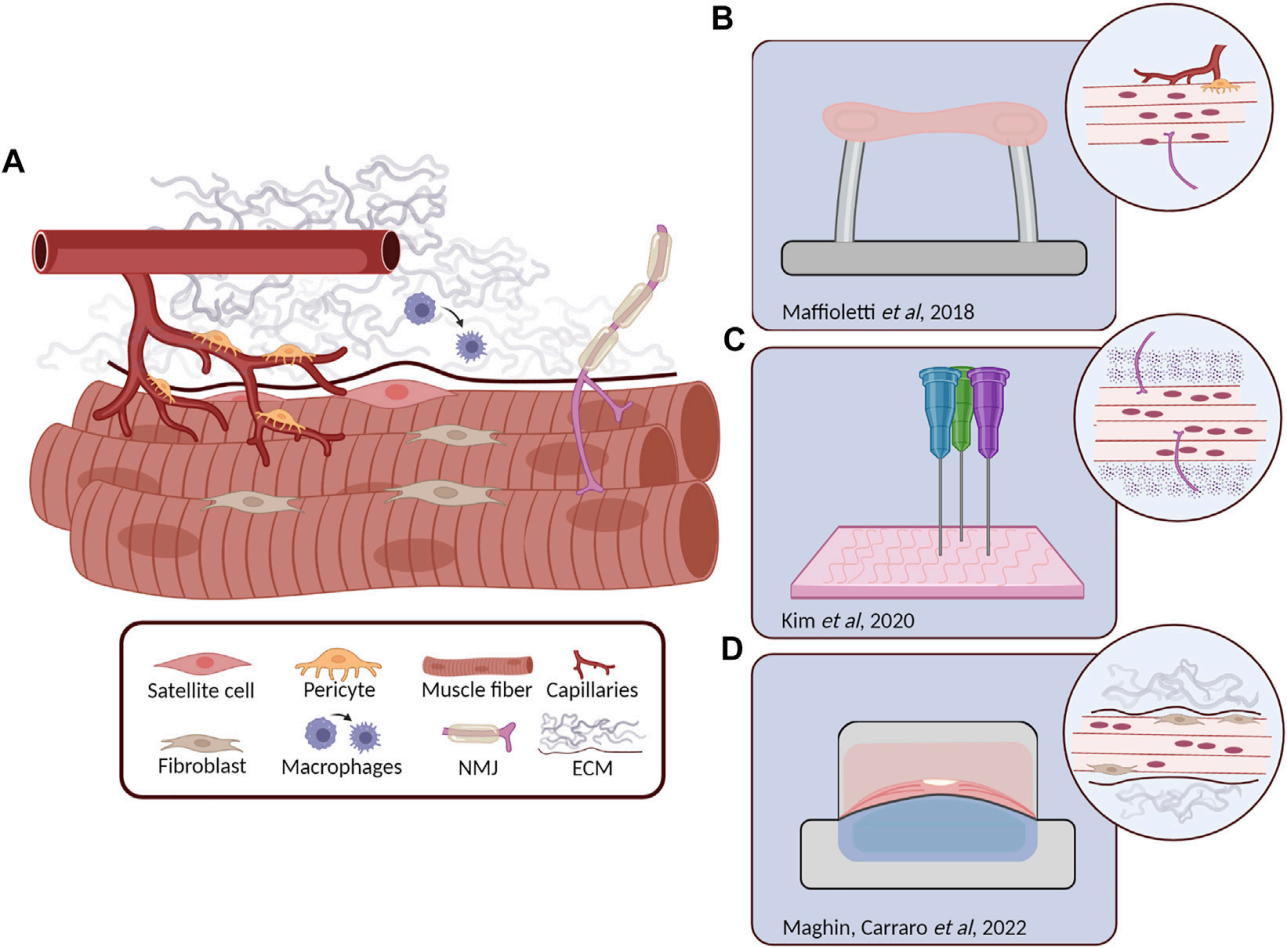
Skeletal muscle (SkM) structure and relevant 3D tissue engineered models. **(A)** Schematic representation of the different cell types composing the SkM tissue. Myofibers are surrounded by ECM proteins, satellite cells are retained underneath the basal lamina, fibroblasts are in close contact with myofibers as the motor neurons that form the neuromuscular junction (NMJ), while pericytes are coming from blood vessels. In the presence of muscle damage, macrophages are recruited to the site of injury where they differentiate from M1 to M2. **(B)** Human induced pluripotent stem (hiPS) derived cells (including myofibers, pericytes, endothelial cells and MN) embedded in a fibrin hydrogel maintained in tension between two pillars. Microscopic analysis revealed the generation of stable 3D constructs with the presence of isogenic myofibers, vascular cells and motor neurons (Maffioletti et al., 2018). **(C)** 3D bioprinting approach for the generation of innervated myofibers following highly precise pattern design (Kim et al., 2020). **(D)** Transversal section of a custom-made bioreactor for the *in vitro* generation of diaphragmatic 3D models, after 14 days of dynamic culture the injected human primary SkM cells and fibroblasts displayed to colonize the proper ECM position, offering a possible solution for the generation of biological patches for the treatment of diaphragm defects (Maghin et al., 2022).

Despite the ambitious and attractive goal to recapitulate *in vitro* every aspect of SkM, tissue engineering offers the possibility to dissect tissue complexity and selectively focus on precise features (Figure 2). Therefore, according to the 3D model characteristics it is possible to effectively study tissue physiology or disease mechanisms by deepening different crucial processes such as tissue maturation, regeneration, or innervation. In this context, there are conditions that must be guaranteed in various cases. If the purpose is the *in vivo* translation, the model should definitely recapitulate healthy SkM characteristics. However, if the goal is to study a muscle disease mechanism, it is essential to obtain *in vitro* a construct as mature as possible. Incorporating different cell types in the 3D model has shown to not only increase *in vitro* construct maturation but also engraftment and survival when tested *in vivo* (Juhas et al., 2018; Sapudom et al., 2021) providing evidence that the raised complexity, if well-orchestrated, is necessary to obtain a reliable and trustworthy model. The presence of aligned myofibers, contractile ability and biomechanical properties are necessary for the *in vitro* reliable validation of human myopathies. Numerous studies, nowadays, are focusing on the translation from healthy to pathological models to obtain *in vitro* platforms for high fidelity drug screening. Currently, DMD is one of the most studied myopathies due to the necessity to overcome the limitations of the use of animal models which are often unable to display severe and human-like disease phenotypes (Aartsma-Rus and van Putten, 2019; Hammers et al., 2020). In recent years, engineered pathological muscle models have been generated showing several specific DMD characteristics such as the reduced myotube size, disrupted calcium handling, membrane instability, and the presence of rare dystrophin revertant fibers, normally absent in 2D models or in the healthy counterpart (Nesmith et al., 2016; Ebrahimi et al., 2021). Since DMD muscle fibers membranes are fragile and susceptible to damage even under physiological stretching, researchers aim to study the electrical properties of the muscle membrane and how using therapeutic agents can change these characteristics. In this regard, an interesting experiment was conducted by Nguyen and colleagues, in which 3D models of DMD muscle tissue displaying electrophysiological aspects similar to *in vivo* samples were treated with poloxamer 188, a possible therapeutic compound, improving their membrane potential and electrophysiological response (Nguyen C. T. et al., 2021).

Specific myopathic characteristics, which may vary from patient to patient, must also be ensured in the production of 3D *in vitro* muscle constructs. In a recent study, hiPS-derived muscle cells from LMNA related dystrophies were encapsulated in fibrin hydrogel and the 3D structures were generated through the tension provided by two opposite pillars. Remarkably, the virtual reconstruction of cell nuclei inside the constructs highlighted significant nuclear aberration, a distinctive feature of LMNA myopathy, demonstrating again the great chance to develop specific and reliable pathological models for possible pathophysiological studies (Maffioletti et al., 2018). These correlations between disease characteristics and the specific 3D model were also highlighted by different works in which the neuronal component was added for modelling diseases that target the adult NMJ (e.g. congenital myasthenia gravis or myotonic dystrophy) (Afshar Bakooshli et al., 2019; Mazaleyrat et al., 2020).

Most myopathies, both inherited and acquired, present chronic inflammation and significant formation of fibrotic tissue as common phenotypes. In 2018 Bersini *et al.* investigated the generation of a fibrotic SkM model by using differentiated human muscle fibers enveloped by a sheath of human muscle-derived fibroblasts and supported by a vascular network (Bersini et al., 2018b). The authors demonstrated the impact of the use of different fibroblastic cell sources. Through the inclusion of DMD-derived fibroblasts (without changing any other cellular component of the system), they observed a significant increase in fibrosis-associated proteins; highlighting once again the importance of choosing the specific cell type to model muscle diseases. Even the tissue regeneration mechanisms, which are the basis of the intrinsic ability of the SkM to recover after major damage, can be clarified and analyzed in a precise way through the use of 3D models. In this regard, the advantage of incorporating macrophages into a 3D SkM model has been reported. Juhas and colleagues, using bone marrow-derived macrophages as immune cell enrichment, demonstrated the complete regeneration of animal-derived 3D muscle after *in vitro* cardiotoxin damage. Although the experiment was pre-clinical work that partially involved the use of animal cells, they showed limited myofibers apoptosis and attenuated pro-inflammatory environment when human-derived macrophages were added to the 3D constructs, supporting the robustness of their model (Juhas et al., 2018).

Although they are not classified as true myopathies, SkM aging and sarcopenia represent a major healthcare challenge since they affect a large proportion of world population. Sarcopenia is a progressive pathology characterized by the loss of muscle mass and strength, prevalent in those over 60 years old (Papadopoulou, 2020). Despite the strong impact of sarcopenia on the quality of life of many elderly people, contractile characteristics of muscle cells from populations of old and young donors have not been extensively studied. Giza and colleagues developed a 3D model of muscle aging to investigate the cellular mechanisms underlying sarcopenia. Through the use of electrical stimulation, they demonstrated that myobundles derived from older sedentary group did not display a synchronous contraction response which was instead present in 3D models obtained with cells from young subjects (Giza et al., 2022) thus providing an aged system to study compound efficacy and toxicity for sarcopenia treatment.

## 5 Conclusion

In the recent decades, tissue engineering has made important steps becoming a branch of medicine that is not only innovative but also reliable and capable of providing concrete therapeutic solutions. If the first clinical applications of *in vitro*-grown tissue (Macchiarini et al., 2008; Elliott et al., 2012) gave credibility and confidence to innovative therapeutic strategies, tissue engineering can also support *in vitro* pharmacological pre-clinical and clinical trials including the prediction of treatment efficacy through the use of patient-specific 3D models. This aspect is of paramount importance, especially in the context of rare diseases, for which there are not enough cases to standardize a therapy, as is the case for several pathologies affecting the SkM tissue. 3D engineered muscles, compared to traditional 2D monolayers, provide longer-term culture, improved myofiber maturation, and the ability to measure functional properties, pathology progression and muscular structure degeneration. The different strategies to enhance construct complexity, including the addition of vasculature, MNs, fibroblasts, and other supporting cell types, allow to generate more biomimetic engineered muscles. At the same time, the choice of precise scaffold material and formulation allows to fabricate constructs with different biomechanical characteristics, mimicking the SkM environment on the basis of the pathology to be modelled. In this context, even the use of ECM derived from (human or animal) myopathic samples can be considered an interesting approach in order to recreate as faithfully as possible the diseased muscular environment.

In conclusion, to obtain *in vitro* a reliable myopathic model, it is necessary to reproduce structures that are as mature and complex as possible which consider not only the different cell types that participate in the development of the disease but also the scaffold that allows cells to recognize any pathological stimuli deriving from the environment. These more physiological 3D models will permit the development of new *in vitro* platforms of SkM tissue for predictive drug screening thereby expanding the spectrum of possible treatments to cure different types of myopathies.

## Supplementary Material

Supplementary Table S1 List of most severe inherited myopathies (MDA, 2022, Orphanet, 2022; Omim, 2022).

Supplementary Table S2 List of most important acquired myopathies (Dalakas, 2009; Pasnoor et al., 2014; Pasnoor and Dimachkie, 2019).

Supplementary Table S3 List of most relevant 3D muscle models.
